# Comparing personalized and population-based models for predicting momentary negative affect in internalizing disorders: A digital phenotyping study

**DOI:** 10.1016/j.nsa.2026.107006

**Published:** 2026-04-28

**Authors:** Leona Hammelrath, Roshan Prakash Rane, Sam Gijsen, Franziska Jüres, Annette Brose, Kerstin Ritter, Kevin Hilbert, Frank Jacobi, Babette Renneberg, Lydia Fehm, Norbert Kathmann, Ulrike Lueken, Christine Knaevelsrud

**Affiliations:** a Department of Clinical Psychological Intervention, Freie Universität Berlin, Berlin, Germany; b German Center for Mental Health (DZPG), partner site Berlin, Potsdam, Germany; c Charité - Universitätsmedizin Berlin, Berlin, Germany; d Department of Psychology, HMU Health and Medical University Erfurt, Erfurt, Germany; e Psychologische Hochschule Berlin, Berlin, Germany; f Department of Clinical Psychology and Psychotherapy, Freie Universität Berlin, Berlin, Germany; g Department of Psychology, Humboldt-Universität zu Berlin, Berlin, Germany; h Hertie Institute for AI in Brain Health, University of Tübingen, Germany; i Tübingen AI Center, University of Tübingen, Germany

**Keywords:** Digital phenotyping, Machine learning, JITAI, Internalizing disorders

## Abstract

Negative affect (NA), encompassing heightened states of sadness, anxiety, and guilt, is a key symptom across a spectrum of internalizing disorders. Recent advancements in digital phenotyping (DP) and machine learning (ML) may enable the automatic detection of short-term fluctuations in NA through digital phenotyping, a prerequisite for Just-In-Time Adaptive Interventions (JITAI). Evidence on the prediction of momentary NA with DP is sparse, but it indicates that personalized ML models are required to account for individual heterogeneities.

This preregistered study is the first to analyze data from the PREACT-digital project, encompassing 242 outpatient subjects diagnosed with internalizing disorders. We examined whether passive sensor data (heart rate, steps, mobility, physical activity) could predict momentary NA, as assessed via ecologically momentary assessments (EMA). Personalized and population-based ML approaches were trained on 19,792 pairs of DP data and NA ratings. We found that personalized ML approaches substantially outperformed population-based models. The best model, however, only marginally exceeded the benchmark, predicting per-person mean NA. Our findings emphasize the need for personalized ML in DP studies. Future efforts could incorporate richer or more raw data streams or test sequential modelling approaches to help clarify whether DP and personalized ML could reliably inform just-in-time, data-driven support for individuals affected by internalizing disorders.

## Graphical abstract

**Figure:**
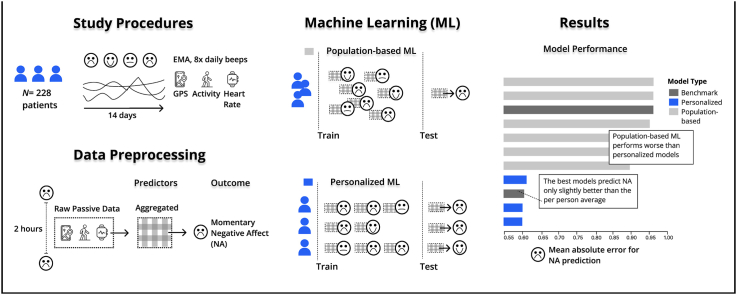

## 1 Introduction

Internalizing disorders, such as obsessive-compulsive disorder, depressive disorders or anxiety disorders, are among the most commonly diagnosed mental health issues worldwide (Global, 2022). A key transdiagnostic component of these disorders is enhanced negative affect (NA), encompassing recurrent experiences of sadness, anxiety, and guilt. NA constitutes a highly complex and heterogeneous construct. It depicts an entanglement of physiological, behavioral, psychological, and contextual concomitants that vary within and between individuals (Adler et al., 2024; Meegahapola et al., 2022; Berkemeier et al., 2024). Cognitive behavioral therapy (CBT) serves as the first-line treatment for internalizing disorders and directly targets NA through interventions like cognitive restructuring and behavioral activation. Yet, its effectiveness is limited, with insufficient response rates reported in around half of patients (Cuijpers et al., 2024). Meanwhile, urgently needed breakthroughs in psychotherapy research remain elusive (Cuijpers, 2024).

With the rapid improvements in innovative sensor technologies and analysis methods like machine learning (ML), there is a rise in attempts to find so-called “digital phenotypes” of mental disorders. Digital phenotyping (DP), also referred to as digital sensing or digital biomarkers, seeks to identify symptom-related behavioral and physiological patterns that can be passively monitored in individuals’ daily lives (Insel, 2017). These data are often captured through sensors embedded in smartphones and wearable devices, and encompass modalities such as heart rate, physical activity and location-based mobility. Evidence suggests that these DP modalities may allow the detection and prediction of negative affect: Heart-rate-derived measures were shown to reflect autonomic activity associated with negative affect (Gullett et al., 2023). Location-based features such as homestay and reduced location variance have been linked to mobility restriction associated with depressive symptoms (Funkhouser et al., 2025)^,^ (Lee et al., 2024). Physical-activity features, including step count, could capture behavioral activation and reduced engagement patterns associated with depressive symptoms (Bizzozero-Peroni et al., 2024).

Beyond monitoring of symptoms like NA, DP has generated considerable interest as a potential source of information for innovative intervention concepts, such as Just-In-Time Adaptive Interventions (JITAI). JITAIs describe interventions that aim to provide the right type and amount of support at the right time by adapting intervention delivery and content to a person's changing internal state and context (Wang and Miller, 2020). In such systems, intervention timing and content are guided by tailoring variables and decision rules. In the present context, predicted momentary NA could serve as a tailoring variable: if elevated or rising NA is detected through passive sensors, predefined decision rules could trigger brief, low-burden support in daily life. For individuals with internalizing disorders, such support could include suggestions for behavioral activation, CBT-informed cognitive reappraisal exercises, or mindfulness and breathing exercises.

Despite the growing interest in leveraging DP for mental health applications (Cuijpers, 2024), only a small number of studies have so far developed or implemented JITAI for NA (van Genugten et al., 2025), or even developed algorithms specifically designed to identify NA based on passive sensor data. (Milne-Ives et al., 2022)^,^ (Khalid and Willis, 2022) Among extant studies, even fewer have addressed moment-to-moment predictions.^17–19^ While approaches like forecasting the next day's or next week's mood (Busk et al., 2020; Cho et al., 2019; Balliu et al., 2024) have their own use case, they are less appropriate for integration into JITAI, as the "just in time" aspect cannot be fulfilled. NA shows daily and context-dependent variability and dispersion in individuals (Kuppens and Verduyn, 2017), which should ideally be captured and addressed in a timely manner. This research gap demands attention, as stable evidence of best-suited features, algorithms, and expected predictive capacities is needed before we can legally and ethically implement them in clinical populations. The current study aims to work towards narrowing this gap: We look to evaluate the predictive capacities of DP for momentary NA and, in doing so, address major challenges in DP research that may have contributed to the shortage of evidence available so far.

First, DP data tend to be extensive, with little organisation, and require careful preprocessing and aggregation in order to serve as useful predictors. As the field is in its infancy, there is no established gold standard for preprocessing DP data, and researchers have many degrees of freedom that may drastically influence predictive performance (Langener et al., 2024) and result in the heterogeneities mentioned above. Until these standards are established, meticulous and transparent documentation, along with justification for all processing steps, is required to increase comparability across studies.

Second, DP data are longitudinal and nested in persons. Unfortunately, there is as of yet no standard or straightforward solution to incorporate subject dependencies into ML models. As a result, previous studies have often neglected to consider this structure adequately and implemented subject-independent models that treat all observations (i.e., aggregated DP features and repeated outcomes) as stemming from the same population (Bai et al., 2021; Han et al., 2024; Lee et al., 2023). These “population-based” approaches cannot depict variations across persons. However, both the perception of NA, as well as the behavioral and physiological manifestations of it, differ within and between individuals. (Adler et al., 2024)^,^ (Berkemeier et al., 2024) By applying population-based, “one-size-fits-all” approaches, these important idiosyncrasies are effectively erased.

A few existing DP studies attempted to model individual variations using “personalized ML” approaches. These approaches can be roughly divided into (1) fully personal models and (2) hybrid models. In (1) fully personal models, the prediction is essentially treated as an N-of-1 modeling task; this means that separate ML models are trained on sequential data of one person. (Berkemeier et al., 2024)^,^ (Wörtwein et al., 2023) It is the most commonly applied “personalized ML” approach and fully embraces the idiographic perspective, but requires sufficient observations to avoid overfitting and to enable reliable generalization to new, incoming data. With regard to implementation, fully personal models are not suitable as they face the “cold-start problem”, i.e., they cannot make reliable predictions for new users. In addition, it was shown that predictive performance can vary strongly between subjects, (Jacobson and Chung, 2020)^,^ (Jacobson and Bhattacharya, 2022) indicating that sufficient accuracy for clinical application cannot be assured. (2) Hybrid approaches aim to balance the strengths of both fully personal models and population-based methods by incorporating person-specific information into the overall model architecture. One way to achieve this is on the feature level, for example, by standardizing DP features on a per-person basis (Kathan et al., 2022) or including latent variables (e.g., age, education) in the feature space. Another strategy (b) involves introducing person-specific parameters to an otherwise population-level model, allowing the model to learn shared patterns while adjusting to each individual's unique characteristics, (Bai et al., 2021)^,^ (Webb et al., 2024) such as Mixed Effect Random Forests (Hajjem et al., 2014) or more advanced methods like transfer learning. (Kathan et al., 2022)^,^ (Yu and Sano, 2020a) By blending individual-level and group-level data, hybrid approaches overcome the “cold-start problem” and reduce the variance in predictive performance found in fully personal models. Both fully personalized and hybrid models were shown to outperform purely population-based approaches in predicting mood (Balliu et al., 2024)^,^ (Han et al., 2024) or depression severity (Kathan et al., 2022)^,^ (Lewis et al., 2023) with DP data, supporting the idea of personalized ML.

To conclude, the limited and heterogeneous landscape of available evidence on algorithms tailored for the passive, moment-to-moment prediction of NA using DP data needs to be addressed before we can proceed with the development of JITAI. The present study intends to contribute to closing this gap, thereby tackling the aforementioned challenges. Following our research objectives, we aim to answer the following questions: (1) Can models trained on entirely passive and contextual features predict momentary NA better than simple benchmark models predicting average NA? (2) Do personalized ML models result in improved predictive performances compared to population-based models? (3) Which personalized ML model yields the best predictive performance?

To answer these questions, we test personalized ML approaches of varying complexity and compare them to population-based counterparts in evaluation scenarios that reflect known users – i.e., data used for model training – versus “new” holdout users. To estimate realistic benefits of personalized ML, we implement both population-based and per-person intercepts (i.e., average NA as benchmarks, as recommended by DeMasi et al. (2017)

As JITAIs are conceptually based on the automatic, unobstrusive identification of critical situations, our algorithms will predominantly rely on passive predictors. Beyond sensor data, a subset of models will be trained on an augmented feature set containing sociodemographic and clinical information with demonstrated relevance to digital phenotyping. These include season (Zhang et al., 2024), weather (Holstein et al., 2024), time of day (Siepe et al., 2024), as well as age, symptom severity and smartphone type (Zhang et al., 2023). We carefully selected these variables to ensure they could be easily assessed within a possible future JITAI framework, thereby maintaining economic validity and facilitating seamless implementation.

## 2 Methods

We strive to increase transparency and reproducibility of our methods as well as comparability with related research. Our study is preregistered on OSF (https://osf.io/54fcj), and the code is published on GitHub (https://github.com/leona-ha/tiki_code). A preprint was published on OSF (Hammelrath et al., 2025a). Detailed methods can be found in our study protocol (Hammelrath et al., 2025b) and in our study-associated OSF directory (https://osf.io/253nb).

### 2.1 Sample and study design

The present study is a subproject of the Research Unit (RU) FOR 5187 “Towards Precision Psychotherapy for Non-Respondent Patients: From Signatures to Predictions to Clinical Utility (PREACT)” (Langhammer et al., 2025). The RU is funded by the German Research Foundation (Deutsche Forschungsgemeinschaft, grant number 442075332) and has received ethical approval from the Institutional Ethics Committee of the Department of Psychology at Humboldt Universität zu Berlin (Approval No. 2021-01) and the Ethics Committee of Charité-Universitätsmedizin Berlin (Approval No. EA1/186/22).

The RU aims to identify predictors of non-response to CBT in internalizing disorders. Individuals seeking CBT in one of four participating university-based outpatient clinics in Berlin, Germany, were informed about the study through flyers and information brochures. Interested patients were invited to an “onboarding” meeting to revise inclusion and exclusion criteria and to provide written informed consent. Inclusion and exclusion criteria are described in more detail in Langhammer et al. (2025) The present study entails the additional requirement of participants having access to an appropriate smartphone.

Participants taking part in our subproject (PREACT-digital) were asked to decide whether they wanted to partake in (1) a shorter version of the study, consisting of a 14-day EMA measurement burst in combination with passive data collection prior to therapy start, or (2) a longer version of the study, consisting of two additional EMA phases after 20 therapy sessions and upon completion of therapy along with parallel passive data collection. In the current study, we will only use passive and EMA data from the first 14-day measurement burst.

For data collection, patients were provided with a state-of-the-art smartwatch (*Withings ScanWatch*), the associated *Healthmate* app, as well as the *TIKI* app, a customized study app developed by a German tech-startup. The *Withings ScanWatch* is able to collect data on sleep, physical activity, and heart rate, and has a comparatively long battery life of around 30 days. The *TIKI* app serves as an interface to the *Withings* API (allowing access to the *ScanWatch* data), collects GPS data, and sends out the EMA questionnaires.

The PREACT study is ongoing, with data collection running until June 2026.

### 2.2 Outcome

We approach the prediction of NA as a regression problem, which more directly aligns with how users gather and make sense of the self-reports. Momentary NA was collected through EMA, eight times a day at approximately 2 h-intervals over 14 days. At each EMA beep, denoting a measurement point, participants completed 17 items from the PANAS-X scale. (Haney et al., 2023)^,^ (Breyer and Bluemke, 2016) Momentary NA was derived from eight items PANAS-X representing four negative affect subscales: sadness (*downcast*, *sad*), fear (*anxious*, *nervous*), hostility (*irritable*, *angry*), and guilt (*ashamed*, *dissatisfied with oneself*). For each item, participants indicated how strongly they felt that emotion “right now” on a 7-point Likert scale ranging from 1 (*not at all*) to 7 (*very much*). The momentary NA score for each beep was calculated as the mean of these eight items, with higher scores indicating greater negative affect at that measurement occasio

### 2.3 Predictors and data preprocessing

Passive data were assessed via (1) the *TIKI* app for GPS data and (2) the *Withings ScanWatch* for all remaining sensor data. Table 1 contains an overview of the features that were used for model training. We applied general and domain-specific preprocessing and aggregation steps depending on the available data format (i.e., raw data vs. data already preprocessed by *Withings*) and sampling frequency. To align passive sensing data with the EMA-based assessment of momentary NA, sensor features were aggregated over the 2-h interval preceding each EMA prompt.

**Table 1:** Overview of predictors and outcomes.

Domain	Construct	Assessment Format	Derived Features	Function
EMA	Negative Affect	8x a day; every 2h ± 0.5h	Mean negative affect per beep	Outcome
Sensing	GPS	Raw; event-based	Distance travelled (km), number of GPS points, minutes at home, minutes spent in transition, minutes spent stationary	Predictor
	Steps	CumSum; event-based	Number of steps	Predictor
	Activity	Boolean; event-based	Minutes in activity states: walking, running, cycling, sleep, rest, active	Predictor
	Heart Rate (PPG)	30-sec mean; 10 min intervals	Mean, min, max, std, hr_zone_resting, hr_zone_moderate, hr_zone_vigorous	Predictor
Time	-	Per Outcome	Assessment hour, time of day, weekday, weekend (binary), season, month	Predictor
Weather	-	Daily aggregates	Apparent temperature (avg), sunshine hours (sum), precipitation hours (sum)	Predictor
Person-Stable	Sociodemographic	Self-report at baseline	Age, employability, smartphone type	Predictor
Person-Stable	Clinical	Self-report at baseline	Somatic problems, prior treatment, current psychotropic medication	Predictor

Abbreviations: EMA = Ecologically Momentary Assessment, GPS = Global Positioning System, PPG = Photopletysmography, avg = average.

Heart rate data were taken from the *ScanWatch* as averages of 30-s intervals sampled every 10 min using photoplethysmography (PPG). They were cleaned by removing non-numeric values, as well as filtering for and removing outliers exceeding the physiological range of 30 to 220 beats per minute (bpm). The cleaned data were then mapped to EMA blocks based on temporal overlap. Within each block, aggregate metrics including mean, minimum, maximum, and standard deviation were computed. In addition to this, time spent in predefined heart rate zones was calculated based on the distribution of heart rate values within each EMA block. We applied the following thresholds for heart rate zones: HR≤60 beats per minute (bpm) resting, HR > 60 bpm <100 bpm moderate, HR≥100 bpm vigorous, in line with guidelines for resting heart rate categorization (Avram et al., 2019).

Step count data were assessed event-based, whenever the *ScanWatch* algorithm detected walking taking place. Step count data were provided as a cumulative sum of steps over the detected sampling period, with timestamps for its start and end. Preprocessing began with the removal of non-numeric and negative values. Durations of step events were calculated from start and end timestamps; entries with zero or negative durations were excluded. Steps per minute were computed. Because minute-level cadences above 200 steps/min are rare even in elite runners (Lyden et al., 2017), values exceeding this threshold were treated as implausible and removed pior to aggregation. The cleaned step data were then mapped to EMA blocks by calculating the overlap between step event timestamps and EMA intervals. Weighted step counts were computed for each EMA block based on the proportion of overlap, and the total number of steps per block was aggregated.

Activity was assessed event-based, i.e., when the *ScanWatch* algorithm detected one out of six activities happening (running, cycling, walking, sleep, rest, active). Invalid entries, such as non-binary activity flags, were removed. All activity samples that overlapped with the start and end timestamps of EMA blocks were aggregated using weighted sums based on the proportion of overlap. This resulted in six features representing minutes spent in the respective activity state during a given 2-h EMA block.

GPS data were collected event-based, whenever the smartphone's operating system detected a location change, and provided in raw format (i.e., longitude-latitude tuples with a timestamp). Accordingly, they required more comprehensive preprocessing steps. Here, we followed the tutorial paper by Mueller et al. (Müller et al., 2021) but adapted its specifications to our event-based sampling format, as this led to high inter-individual variability in the number of acquired GPS data.

To transform the raw GPS traces into interpretable mobility features (e.g., *time spent at home*, *number of distinct places visited*), we first needed to aggregate spatially adjacent points into meaningful locations—a step referred to as clustering. Reliable clustering requires a minimum density of location samples, so we excluded participants who contributed fewer than 50 GPS points during a measurement burst. Several thresholds were tested; a cutoff of 50 offered the best balance between retaining as many participants as possible and ensuring that the resulting clusters (and hence derived features) were robust. Distances between consecutive GPS points were computed using the Haversine formula as follows: distance = sin2(Δφ/2) + cos φi cos φj sin2(Δλ/2), where φ and λ correspond to the latitude and longitude. Speeds were derived by dividing the calculated distances by the time differences between consecutive points. GPS points were classified as stationary if their speed was below walking speed (i.e., 1.4 m/s) and the distance between consecutive points was less than 150 m. Stationary GPS data were clustered using the DBSCAN algorithm (Schubert et al., 2017), with normalized min_samples (i.e., 3% of a person's total number of GPS points) and epsilon = 100/6371000. As the home cluster, we selected the cluster where (1) a person spent at least four nights (i.e., between 8 p.m. and 6 a.m.) and (2) at least 50% of their total assessed nights. As this left us with many participants not having a home cluster, likely due to the event-based assessment format not collecting data when no change of location took place, we implemented a fallback home cluster, i.e., the most frequently visited cluster per person. Following this, we calculated the following features after mapping their timestamps to the respective EMA blocks: *number of GPS points* as cumulative sum during a block, *distance travelled* as the sum of the distances for all GPS points in that EMA block, *minutes at home* by summing the durations for GPS points belonging to the home cluster during a Block, *transition minutes* by summing the durations for GPS points classified as non-stationary and *stationary minutes* by summing the durations for GPS points classified as stationary.

Contextual features incorporate weather and time features. Daily weather information was sourced from *Openmeteo* (https://open-meteo.com/) for the coordinates of Berlin Alexanderplatz (52.521992, 13.413244). We referenced the average apparent temperature, the sum of sunshine hours, and the sum of precipitation hours for assessment days. Time features were derived from the time at which the EMA assessment was completed and included assessment hour, time of day (early morning, morning, afternoon, evening, night), season (spring, summer, fall, winter), month, weekday, and weekend (binary).

Person-stable information was assessed during an onboarding appointment at the outpatient clinic. To maintain economic validity and implementability, we considered only simple sociodemographic and self-report clinical measures. We included the metrics of age, employability (“Are you currently fit for work?”, binary), somatic problems (“Do you have physical illness?”, binary), smartphone type (*iPhone, Android)* as well as current psychotropic medication (binary) and previous psychotherapeutic or psychiatric treatment history (no prior treatment, prior psychotherapeutic treatment, prior inpatient treatment) as proxies of symptom severity.

In total, we included 28 passive and contextual time-varying features and six person-stable features as predictors.

### 2.4 Missing data handling

We excluded participants who had fewer than seven days with at least four completed beeps (i.e., 25% of measurements) to maintain comparability to previous studies (Siepe et al., 2024) and to ensure that daily and weekly fluctuations could be depicted. Furthermore, patients were excluded if they had fewer than 50 GPS points in total during the first assessment phase to ensure that GPS features could be calculated with sufficient reliability.

Passive data streams were marked as missing if no datapoint was available in the 2-h blocks preceding a beep. For features assessed with an event-based sampling scheme (GPS-derived data, physical activity categories, steps), missing values were set to a fixed value if it could reasonably be assumed that they were missing due to the absence of an event (i.e., location change, detected walking). This value was set as 0 for *distance travelled*, *number of GPS points*, *minutes spent in transition*, *steps,* and all physical activity values, and 120 for *time spent at home* and *time spent stationary.* We added further checks to ensure that data were not missing completely at random (MCAR), i.e., due to patients not wearing the *ScanWatch* or common technical problems causing interruptions of GPS data collection. For missing GPS data, we checked whether a person indicated being “travelling” withing the last 2 h as part of the EMA-based situation assessment. If that condition was met we assumed that the person moved and should accordingly have provided GPS data. If this condition was met in more than 50% of an individual's available EMA blocks, they were excluded from further analysis. Rows featuring MCAR were marked and imputed using knn (see section *Model Pipeline*).

For wearable data (i.e., activity type, heart rate, steps), we checked whether all other *ScanWatch*-derived features were also missing for that measurement block, indicating patients not wearing the watch. These entries were imputed using *knn* (see section *Model Pipeline)*. To conclude, we re-evaluated whether participants still met the condition of a minimum of 7 days with at least 4 completed beeps and excluded those who did not meet this requirement.

### 2.5 Modeling approaches

Supplementary Table 1 provides an overview of all model types and hyperparameters applied. We implemented population-based, as well as personalized ML approaches to predict NA at time *t* based on passive and contextual features at *t-2h*.

**Purely population-based:** In population-based models, every sample is treated as coming from the same population. In terms of implementation, these can be seen as a “one-size-fits-all” solution, aiming to find a single function that best explains variation across the entire dataset. Consequently, they could potentially be directly applied to new samples from both known or new users. For population-based models, we chose a range of linear and non-linear machine learning approaches that are commonly used in psychology (Kovač et al., 2024).

a.) **Random Forest Regressors** (RF): Random Forests (Breiman, 2001) combine multiple decision trees to improve predictive performance and reduce overfitting. Each tree is trained on a random subset of the data and a random subset of the features; the final prediction is typically an average across all trees. Random Forests are robust to outliers and can model complex nonlinear relationships.
b.) **Linear Regression** (LR): Linear regression is a fundamental statistical technique used to model the relationship between a dependent variable and one or more independent variables. It assumes a linear relationship and estimates parameters that minimize the sum of squared errors. Linear regression is straightforward and fast to train (Montgomery et al., 2021).
c.) **Feed Forward Neural Network** (FFNN): A Feed Forward Neural Network is a deep learning architecture, composed of layers of nodes (neurons) in which connections only propagate forward from input to output. This model learns via backpropagation, by adjusting weights in each fully connected layer to minimize prediction error. FFNNs can capture nonlinear relationships and interactions among predictors but may require large datasets and careful tuning of hyperparameters (e.g., number of layers, learning rate, activation functions). (Deep Learning) The FFNN key components implemented in our study are:
  (1) Input Layer: Receives a vector of aggregated sensor data (e.g., 2-h averages).
  (2) Hidden Layers: One or more fully connected layers, each applying a linear transformation (weight matrix + bias) followed by a non-linear activation (e.g., ReLU).
  (3) Output Layer: A linear regression node outputs a numeric prediction reflecting

NA.

(1) **Feature-based personalization via person-stable features:** Population-based baseline models were trained on an augmented feature set including person-stable (PS) attributes (e.g., age) in order to capture between-user variability. This low-key personalization strategy could easily be implemented for new users by collecting their stable information beforehand.
  a.) **RF + PS:** person-stable features were added to the feature set alongside passive data.
  b.) **LR + PS:** person-stable features were added to the linear model alongside passive data.
  c.) **FFNN + PS:** person-stable features were concatenated with passive data before entering the neural network via the input layer.

Subsequent approaches include model-internal representations of individuals by learning intercepts (MERF) or person embeddings (FFNN + Embedding).

(2) **Personalization via Mixed-Effect Random Forests** (MERF): Mixed-Effect Random Forests (MERF), introduced by Hajjem et al.,32 combine the flexibility of Random Forests with the personalization capabilities of linear mixed-effect models. They were successfully applied to predict depression severity based on DP data (Lewis et al., 2023). Similar to fixed effects in linear models, the Random Forest component learns a global, non-linear function by pooling samples across clusters. Meanwhile, person-specific random intercepts are learned by training a linear mixed model, adjusting global predictions to account for individual differences, thereby producing personalized predictions. For new users, the model would require an initial set of data (e.g., a few days of observation) to accurately estimate their random intercepts.
  (a) **MERF:** Learns a global Random Forest plus random intercepts for each individual.
  (b) **MERF + PS:** Extends MERF by additionally incorporating person-stable features.
(3) **Personalization via Neural Networks with Person-specific embeddings**: Entity embeddings map high cardinality categorical variables (e.g., participant IDs) into dense, continuous vectors, revealing latent similarities among categories that may not be captured by traditional feature engineering (Guo and Berkhahn, 2016). With this approach, we treated each participant ID as a high cardinality categorical variable, assigning a person embedding that captures latent traits beyond person-stable information. These learned embeddings were then concatenated with aggregated 2-h sensor data and fed into neural networks. For new users, this model would require a training phase to update the embedding layer as sufficient data from the new user accumulates. To leverage these person embeddings, we implemented them into the FFNN architecture.
  (a) **FFNN + Embedding:** In the embedding layer, each participant ID was mapped to a lower-dimensional (i.e., 32, 64) embedding vector. The embedding was concatenated with the sensor features, then projected to the FFNN input dimension.

### 2.6 Model Pipeline

**Fig. 1:**
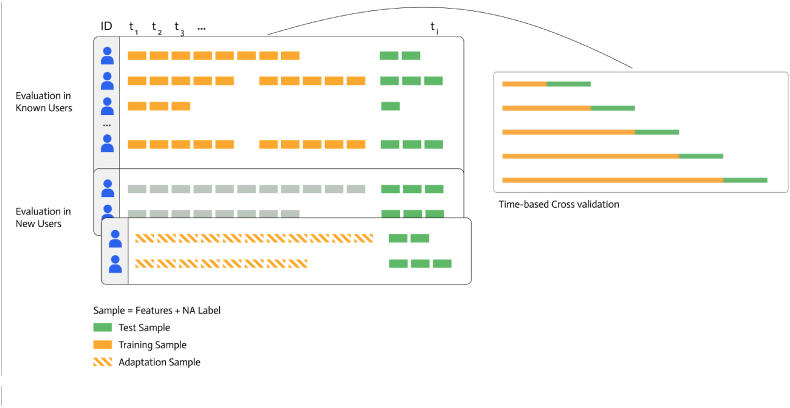
Model evaluation scenarios.

Our model evaluation pipeline consists of (a) an outer user-based split, selecting 10% of the participants as a holdout set, and (b) inner time-based splits, wherein data from the remaining 90% of participants is divided into (1) a training set containing the first 80% of samples per participant and (2) a test set containing the remnant 20% of samples per participant. An overview of our evaluation scenarios is depicted in Fig. 1.

As a first step, we created a 10% holdout sample (user split), stratified by the number of available samples per person (i.e., EMA labels with associated sensor data). This holdout set was used to evaluate how well the model generalizes to completely new users that were never seen during training or model tuning. The remaining 90% of the sample was used to train our model and to evaluate how the model performs for new samples by known users. Here, we applied a time-based split, in which we assigned the chronologically first 80% of data per user to the training set and the remaining 20% to the test set. Hyperparameter tuning was conducted using grouped, time series cross-validation with five splits. Per participant, data were divided chronologically into roughly equal chunks. In the i-th fold, the model is trained on all earlier chunks and validated on the i-th chunk. As the folds progress, the training set grows in an expanding window fashion, which mimics a real-world scenario wherein new data becomes available over time. The first fold was skipped to prevent a situation where the training set is empty, resulting in 4 folds.

For the holdout sample we sought to reproduce two possible implementation scenarios: (1) all models were tested on the final 20% of data from each holdout user, *without any adaptation*, to simulate performance in new users who have no historical data; (2) for the FFNN + Embedding pipeline, we simulated a partial fine-tuning phase, in which the first 80% of each holdout user's data was utilized to update only the embedding layer. All remaining model layers were frozen, ensuring that the globally learned parameters stayed fixed while user-specific adjustments happened solely within the embeddings. We did not apply holdout adaptation for the MERF model as this would have required fully retraining its Random Forests component, making the adaptation procedure inconsistent with the partial adaptation scheme used in FFNN + Embedding.

A k-Nearest Neighbors (k-NN) imputer was first fitted onto the training data to learn the underlying structure of the missing GPS features. The learned imputation model was then used to fill in missing values in both the time-based and user-based holdout sets. Categorical features were one-hot encoded. Continuous features were standardized using Min-Max Scaling based on training data statistics. This scaling was then applied to the holdout sets (i.e., time-based holdout and all samples from holdout users). Right-skewed continuous variables were log-transformed before scaling. Features with zero or near-zero variance were removed. Hyperparameters were tuned using *scikit-learn*'s GridSearchCV.

Predictive performance was evaluated using the mean absolute error (MAE) on the test data. We further calculated the root mean squared error (RMSE) and R^2^ for comparability with other studies. We included two intercept-only baseline models: The global intercept model predicts the grand mean of the outcome computed over the entire training set, while per-person intercepts predict the mean outcome per participant, computed from their training samples.

## 3 Results

### 3.1 Sample and data quality

In total, 403 participants provided data for the first assessment phase. During quality control, 123 participants had to be excluded due to an insufficient number of completed beeps. A further 32 participants were excluded due to insufficient total amounts of GPS samples. 2 participants had to be excluded due to missing GPS predictors in more than 50% of available beeps. After removing rows with missing values in all passive features, 6 individuals no longer fulfilled the inclusion condition (i.e., a minimum of 4 completed beeps on at least 7 days) and were excluded. This left us with N = 242 participants for analysis. Table 2 presents sample characteristics for included and excluded participants. Included participants showed a higher proportion of employable individuals than excluded participants (81.0% vs. 72.0%, *p* = .048). No other statistically significant differences were observed between groups in the examined sociodemographic or clinical variables.

**Table 2:** Sample description comparing included, quality-assured vs. excluded subjects.

Variable	Overall	Subjects not included	Subjects Included	p-value
***N***	403	32.5 (11.3)	33.4 (10.4)	
**Age, mean (SD)**	33.0 (10.8)	45 (28.0)	46 (19.0)	.440
**Employable, n (%)**		116 (72.0)	196 (81.0)	
No	91 (22.6)	64 (39.8)	114 (47.1)	.048
Yes	312 (77.4)	97 (60.2)	128 (52.9)	
**Smartphone Type, n (%)**		57 (35.4)	90 (37.2)	
Android	178 (44.2)	49 (30.4)	64 (26.4)	.176
iPhone	225 (55.8)	55 (34.2)	88 (36.4)	
**Previous treatment, n (%)**		32.5 (11.3)	33.4 (10.4)	
No treatment	147 (36.5)	45 (28.0)	46 (19.0)	.682
Inpatient therapy	113 (28.0)	116 (72.0)	196 (81.0)	
Outpatient therapy	143 (35.5)	64 (39.8)	114 (47.1)	
**Psychotropic medication, n (%)**				
No	262 (65.0)	105 (65.2)	157 (64.9)	1.00
Yes	141 (35.0)	56 (34.8)	85 (35.1)	
**Diagnosis, n (%)**				
PD	29 (7.2)	12 (7.5)	17 (7.0)	.754
MDD	171 (42.4)	65 (40.4)	106 (43.8)	
GAD	35 (8.7)	15 (9.3)	20 (8.3)	
OCD	54 (13.4)	23 (14.3)	31 (12.8)	
PTSD	20 (5.0)	7 (4.3)	13 (5.4)	
SAD	75 (18.6)	29 (18.0)	46 (19.0)	
SP	14 (3.5)	6 (3.7)	8 (3.3)	
Missing	5 (1.2)	4 (2.5)	1 (.4)	
**Somatic problems, n (%)**				
No	224 (55.4)	89 (55.3)	133 (55.0)	1.00
Yes	180 (44.6)	72 (44.7)	109 (45.0)	

**Note.** Values are presented as *M* (SD) or *n* (%). *P* values reflect comparisons between included and excluded participants using Welch's two-sample *t* tests for continuous variables and chi-square tests for categorical variables. Abbreviations: MDD, major depressive disorder; GAD, generalized anxiety disorder; SAD, social anxiety disorder; PD, panic disorder; OCD, obsessive compulsive disorder; PTSD, post-traumatic stress disorder; SP, specific phobia; SD, standard deviation.

After preprocessing, an average of 85.60 ± 17.00 beeps remained per participant, with a minimum of 48 and a maximum of 116 beeps. In total, this left us with 19,792 samples containing passive sensor data aggregated across 2 h and subsequent NA ratings. Step count was missing in 6.36% of samples. In 520 of those samples, other smartwatch features were missing as well, which were imputed using k-NN. In all remaining cases, other smartwatch features were available, indicating the absence of an event (i.e., walking), and were thus set to 0. The same applied to all other activity categories, which had a missingness of 58.46% across samples. 520 of those samples were imputed, as all other smartwatch features were missing as well. The leftover features were set to 0. GPS data were missing in 25.27% of samples. In 911 of those samples, the person indicated travelling and were imputed; the rest were set to 0. Heart rate features were missing in 3.40% of samples and were imputed.

**Fig. 2:**
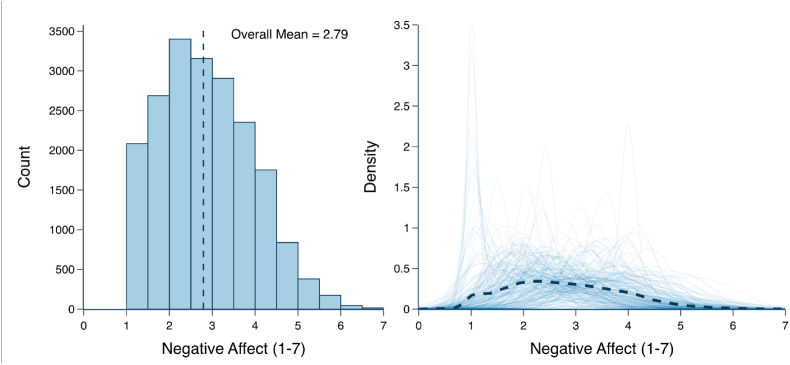
**Distribution of Negate Affect Ratings.** The left side depicts the distribution of average negative affect (NA) ratings across all participants and beeps. Higher ratings indicate higher NA. The right side depicts the per-person density of NA ratings, with the black dotted line showing the average density of NA.

Fig. 2 shows two complementary visualizations of NA scores. The left panel shows an aggregated histogram of scores across all participants, while the right panel overlays individual kernel density estimates that capture each participant's negative affect ratings over time. The overall mean NA score was 2.89 ± 1.09, and the distribution was slightly right-skewed, indicating that most ratings fall on the lower end of the scale and very high ratings occur only infrequently. Moreover, the shapes of the individual density curves reveal substantial intra-individual variability, suggesting that while many participants generally report low levels of NA, their moment-to-moment experiences can vary considerably.

### 3.2 Predictive analysis

Out of 242 participants, 24 users, supplying a total of 1961 samples, were randomly assigned to the holdout set as part of the user-based split. Of the remaining dataset, 3656 samples were assigned to the test set as part of the time-based split, leaving 14,175 samples for model training.

*Time-based holdout:* Table 3 summarizes the performance of all modeling approaches on the time-based holdout set. Overall, MAE ranged from .596 to .943 on the 1–7 NA scale, indicating that predictive accuracy varied substantially across model classes. The best performance was achieved by the MERF model, which yielded the lowest MAE (.596) and RMSE (.795) and the highest explained variance among all models (R^2^ = .520). However, its advantage over the simple per-person intercept baseline (MAE = .600, R^2^= .512, RMSE = .802) was very small. Similarly, the FFNN + Embedding model performed comparably to the per-person intercept baseline (MAE = .609, R^2^ = .516, RMSE = .799), but did not improve upon it. Taken together, these findings suggest that explicitly modeling person-specific information was important for prediction, while the added value of more complex modeling approaches remained limited.

**Table 3:** Model performance in time-based holdout data, consisting of the last 20% of samples per participant.

Model	MAE a	R^2^^b^	RMSE c
Global Intercept	.943	−.003	1.149
**Per Person Intercept**	**.600**	**.512**	**.802**
LR	.937	.004	1.146
RF	.932	.012	1.141
FFNN	.936	.008	1.143
LR + PS	.921	.035	1.128
RF + PS	.846	.177	1.042
FFNN + PS	.878	.116	1.079
**MERF**	**.596**	**.520**	**.795**
MERF + PS	.605	.507	.806
FFNN + Embedding	.609	.516	.799

Bold numbers indicate the best-performing models.

Abbreviations: LR = linear regression; RF = random forests; FFNN = feed forward neural network; PS = person-stable features; MERF = mixed effect random forests.

a Mean absolute error (MAE): lower is better.

b Coefficient of determination (R^2^): higher is better.

c Root mean squared error (RMSE): lower is better.

Among the global models without person-specific extensions, performance was consistently poorer. The global intercept model performed worst overall (MAE = .943, R^2^ = −.003, RMSE = 1.149), and the standard LR, RF, and FFNN models yielded only minimal improvements over this baseline. Adding person-stable features (PS) improved performance for all three approaches, particularly for RF (MAE = .846, R^2^ = .177, RMSE = 1.042) and FFNN (MAE = .878, R^2^= .116, RMSE = 1.079), although these models still remained clearly below the per-person intercept and MERF approaches. Notably, adding person-stable features to MERF did not further improve performance (MERF + PS: MAE = .605, R^2^ = .507, RMSE = .806). Overall, the pattern of results indicates that most predictive signal was captured by stable individual differences rather than by more complex feature-based modeling.

*New User Holdout:* Table 4 shows the results for the 24 participants who were not seen during training. Overall, MAE values ranged from .682 to .934 on the 1-7 NA scale. The per-person intercept achieved the lowest MAE (.682) and lowest RMSE (.885), while FFNN + Embedding performed very similarly (MAE = .693, RMSE = .885) and showed the highest R^2^ (.293). However, none of the models clearly outperformed the per-person intercept baseline. Most other approaches showed only small improvements in MAE over the global intercept (MAE = .874), and many yielded near-zero or negative R^2^ values, indicating limited generalization to unseen users. Adding person-stable features did not improve performances. Overall, these findings suggest that predicting NA for entirely new users remained challenging and that more complex models offered little benefit beyond simple person-specific baselines.

**Table 4:** Model performance in new user holdout data.

Model	MAE a	R^2^^b^	RMSE c
Global Intercept	.874	−.043	1.075
Per Person Intercept d	.682	.287	.885
LR	.866	.004	1.050
RF	.867	−.025	1.056
FFNN	.866	−.010	1.060
LR + PS	.934	−.122	1.145
RF + PS	.931	−.127	1.117
FFNN + PS	.928	−.213	1.117
MERF	.857	.004	1.050
MERF + PS	.924	−.116	1.112
FFNN + Embedding d	.693	.293	.885

Abbreviations: LR = linear regression; RF = random forests; FFNN = feed forward neural network; PS = person-stable features; MERF = mixed effect random forests.

a Mean absolute error (MAE): lower is better.

b Coefficient of determination (R^2^): higher is better.

c Root mean squared error (RMSE): lower is better.

d Per Person Intercept and FFNN + Embedding were adapted in holdout users based on the first 80% of their data.

## 4 Discussion

The present study aimed to extend the sparse and inconsistent evidence on DP-based prediction of momentary negative affect. To assess the predictive value of passive and contextual features in forecasting NA, we compared a range of models trained on these features to a simple baseline model that calculates the average NA for the population or individual. Beyond that, we evaluated the added value of adjusting model pipelines to expected inter- and intra-individual variances by implementing different personalized ML approaches and comparing them with their population-based counterparts in known and new users. We found that personalized ML models outperformed population-based models. At the same time, however, the simple per-person mean benchmark performed similarly to the best-performing models. Thus, a substantial part of the predictable variance in momentary NA may lie in stable individual differences in average affect level and the advantage of personalization in the present study may have come from capturing person-specific baselines rather than modelling moment-to-moment fluctuations around those baselines. This finding is critical, and emphasizes the need of appropriate benchmark models to interpret predictive performance. In terms of clinical implementation, our model performances suggest that DP data have limited capacity to support precise and automatic real-time intervention triggering.

Our first research question targeted the general feasibility of predicting momentary negative affect with passive data. To find an answer, we evaluated whether models trained on 2-h aggregates of passive sensor data and contextual concomitants outperformed benchmark models in predictive accuracy. Among known participants, all models, except the FFNN, both with and without person-stable features, achieved better performance than the benchmark model predicting the mean NA across all samples. MERF, as well as FFNN combined with person-specific embeddings, performed slightly better than the benchmark predicting per-person average NA, but accomplished similar overall performances. Incorporating user-specific adaptations - through either embedding-based personalization or mixed effects modelling - can capture individual variability to some extent, but the additional predictive gain over a simple per-user mean might be marginal. Not surprisingly, the models also did not generalize well to new users. Our second and third research questions addressed the added value of model personalization. We compared personalization strategies of different complexities to population-based equivalents. In line with prior work (Amin et al., 2025), we found that all personalization strategies improved predictive performance compared to purely population-based models, underscoring the need to personalize predictive modelling. The best performance was reached by FFNN + Embeddings, closely followed by MERF. For time-based scenarios, adding person-stable features (PS) benefited population-based models (RF, LR, FFNN). Still, it did not improve MERF, presumably because MERF's random effects already accounted for much of that user-specific signal. In the new-user holdout, models generally performed worse, indicating limited generalization. FFNN + Embeddings, with embeddings finetuned in the holdout sample, performed best, but did not outperform per-person average NA.

In the following sections, we locate our findings within the current literature and critically discuss the impact of feature selection, outcome definitions, modelling approaches, and implementation scenarios.

The concept of JITAI relies on passive data to automatically identify critical affective states. Our results, however, question whether passive sensor data can meet this prerequisite, or whether affect fluctuations are mainly driven by stable interindividual differences.

Evidence for the passive prediction of momentary affect is sparse and mixed, aggravating the categorization of our findings. Cai et al. (2018) explored passive prediction of state affect in a 2-week study of 220 college students. They defined “momentary” features as contextual features extracted from the 1-min window preceding each EMA prompt and compared generalized models trained on all participants' data with personalized models trained on data from the respective participant only. Across random forest, SVM, and XGBoost models, personalized models outperformed generalized models; the best performance for negative affect was reported for the personalized SVM using momentary features (RMSE = 12.82 on a 100-point scale). Jacobson and Chung (2020) predicted hour-to-hour changes in depressed mood among undergraduates with clinically elevated depression, using passive phone-sensor data augmented by sporadic heart rate readings. They combined a population-based model (XGBoost) with per-person model weight adjustments, finding a mean correlation of approximately .59 between the predicted and observed hourly moods. Although forecasts varied among individuals, most participants' mood fluctuations could still be modelled successfully. However, both studies did not include a simple benchmark such as the person-specific mean affect. Thus, it remains unclear whether passive features provided incremental predictive value beyond a person's average affect level. Li and Zhang (2025) investigated affective state recognition in everyday contexts using the DAPPER dataset, which included five days of continuous multi-modal, wearable and EMA recordings from 88 subjects. Predictors included galvanic skin-response, accelerometer and PPG data that were, in contrast to our study, were available in raw format. Their model included (2) automatic deep learning-based feature extraction from the 30-min window preceding each EMA beep, and (2) a Transformer that was, in contrast to our modelling approaches, able to capture long-term temporal dependencies and cross-feature interactions. Benchmarking against traditional models (e.g., Random Forests), their model achieved a classification accuracy of 71.5% for predicting positive versus negative affect.

In summary, the studies above found that, in contrast to our study, passive data were informative for predicting momentary affect. As they did not include a per-person-average, or comparable, benchmark, we cannot directly compare them to our results. Nonetheless, they identify certain issues that may have contributed to the limited incremental value of passive data beyond person average NA and could be addressed in the future.

The first issue concerns the granularity, quality and information value of our features. To preserve patients’ privacy and minimize the risk of recognition as study participants, we opted to use commercial wearables for data collection. Except for GPS, passive signals were therefore available only as *Withings*-derived summaries (e.g., step count vs. raw accelerometer), limiting feature derivation and introducing a potential source of noise (Onnela, 2025). Research-grade sensors provide raw, high-resolution data, enabling advanced preprocessing and deep learning–based feature learning to enhance accuracy. (Li and Zhang, 2025)^,^ (Rykov et al., 2024)

GPS was available in raw format but was assessed event-based, unlike most previous studies, i.e., when the smartphone's operating system detected movement. This approach was chosen deliberately to reduce battery drain and thereby alleviate participant burden. Still, it led to heterogeneous data distributions across participants and forced us to deviate from typical preprocessing pipelines (Müller et al., 2021). Additionally, our research app experienced technical difficulties with GPS data collection, resulting in higher missing-data rates than with wearable-derived data sources. Beyond that, momentary affect is strongly shaped by the immediate situational context (de Vries et al., 2021), such as social interactions and ongoing activities. Although such contextual information was assessed in the broader study (i.e., using EMA items assessing social and situational context), we intentionally excluded it from the present models because our aim was to evaluate the predictive value of passive features only. This design choice increased ecological and practical relevance for low-burden JITAI applications, but it may have constrained predictive performance.

Secondly, our ML approaches did not explicitly model temporal dependencies in DP features and NA ratings. This was intended, as we deemed it necessary to first evaluate the added value of incorporating person-level data for the prediction of momentary NA. Nonetheless, DP features also exhibit high intra-individual variability over time (Bögemann et al., 2024). A simple solution here would be the implementation of longer, rolling aggregation windows, (Jacobson and Chung, 2020)^,^ (Sun et al., 2023), i.e., utilising data from the 4 h preceding a NA rating. Another option is the integration of sequential components into modelling approaches. For instance, we could process the sequential sensor data using neural network layers designed for temporal modelling, such as LSTMs or Transformer encoders with positional encoding. (Kathan et al., 2022)^,^ (Yu and Sano, 2020b) These layers would learn the evolution of sensor readings over time, capturing short- and long-term dependencies. Then, by concatenating the output of these temporal layers with the person-specific embeddings, our model could jointly learn both the individual's baseline behaviour as well as how their sensor patterns change over time. More research is needed to determine whether increased model complexity could reduce predictive errors.

## 5 Strengths and limitations

Beyond the aforementioned issues, our study has some limitations that must be considered. Firstly, we had to exclude almost half of our sample due to an insufficient amount of completed EMA beeps and other data quality issues. Excluded participants had significantly lower rates of employability, which could indicate higher symptom load. This has important implications for the real-world feasibility of digital phenotyping in clinical care. Approaches such as the one studied here depend on repeated EMA completion, continuous sensor availability, and sufficiently high technical data quality over time. In routine implementation, these requirements may be difficult to meet without substantial participant engagement, technical reliability, and ongoing support. Although this pattern is in line with other DP studies, it highlights a central implementation challenge for DP-based approaches in routine care. With regard to data preprocessing, we faced the need to make various micro-decisions (e.g., minimum threshold for completed EMA beeps), which may gravely influence outcomes (Niemeijer et al., 2022) and should ideally be met using methods such as multiverse analysis. To handle missing data, we implemented a relatively simple imputation technique to improve computational efficiency. As shown by Rashid et al. (2020), more elaborate imputation techniques are another option to increase predictive performance. Finally, relationships between NA and DP might differ across diagnoses (i.e.,depression and obsessive compulsive disorder). Our transdiagnostic approach, although ecologically valid, might have obscured disorder-specific patterns and attenuated the predictive signal.

The strengths of our study should not remain unmentioned: First, our sample is more comprehensive than in most past and present DP studies and consists of patients with both a diagnosis categorised as internalizing and a willingness to start psychotherapy. This sample represents a conceivable target group for a JITAI addressing negative affect, increasing the ecological validity of our study. Second, within this clinical sample, we implemented a comparably high frequency of EMA assessments and thus NA outcome labels, allowing us to capture and predict momentary NA with DP data. Third, we systematically evaluated the added value of personalization and model complexity. We implemented fair benchmark models to assess the added value of DP data realistically. Moreover, we considered different implementation scenarios by testing the models not only with known users but also with new users. As the fields of personalized ML and DP are in their infancy, our research adds valuable insights for future studies in the field.

## 6 Conclusions

The current study aimed to pave the way towards JITAI for negative affect by analysing the potential of passive data for predicting momentary NA. In a comprehensive sample of patients diagnosed with internalizing disorders, we were able to show that NA fluctuates significantly within and between persons. However, DP features were not able to explain these momentary fluctuations in NA beyond stable person-specific baselines. This finding, in line with high attrition rates due to insufficient data quality tempers hope for the implementation of DP into JITAI. To proceed in this area and keep up with major, data-rich companies, we need more high-quality studies employing large clinical samples. The field of DP in mental health in is in its infancy, but there is a momentum that is unlikely to expire too soon: sensor technology is constantly improving, as is the type and quality of data sensors can collect. Parallel to this, Large Language Models facilitate the implementation of complex ML architectures and the preprocessing of DP data in clean, readable code, thereby increasing reproducibility. Thus, it remains to be seen whether continuous passive sensing can be employed to detect critical states, e.g., negative affect, and hence bring about the desired improvements in the treatment of internalizing disorders.

## Code availability

The underlying code for this study is available on GitHub at the following link: https://github.com/leona-ha/tiki_code.

## Author contributions

Conceptualisation, L.H. and R.R.; Methodology, L.H., R.R., S.G., A.B., K.R., and K.H.; Formal Analysis, L.H. and R.R.; Data Curation, L.H. and F.J.; Writing – Original Draft, L.H.; Writing – Review & Editing, L.H. ; Visualisation, L.H.; Supervision and Project Administration, A.B. and C.K.; Funding Acquisition, U.L., C.K., K.R., K.H., F.J., B.R., L.F,. and N.K. All authors contributed to the critical revision of the manuscript and approved the final version.

## Funding statement

Funded by 10.13039/501100001659Deutsche Forschungsgemeinschaft, grant number 442075332.

## Declaration of competing interest

The authors declare that they have no known competing financial interests or personal relationships that could have appeared to influence the work reported in this paper.

## Data Availability

The datasets generated and analysed in the course of the current study will not be publicly available while the study is ongoing (until 06/2026). After the study is completed, they can be made available upon reasonable request.

## Abbreviations

API: Application Programming Interface
CBT: Cognitive Behavioral Therapy
DBSCAN: Density-Based Spatial Clustering of Applications with Noise
DP: Digital Phenotyping
ECG: Electrocardiogram
EMA: Ecological Momentary Assessment
FFNN: Feed Forward Neural Network
GPS: Global Positioning System
HR: Heart Rate
JITAI: Just-In-Time Adaptive Intervention
knn: k-Nearest Neighbors
LSTM: Long Short-Term Memory
LR: Linear Regression
MAE: Mean Absolute Error
MERF: Mixed-Effects Random Forest
NA: Negative Affect
OCD: Obsessive-Compulsive Disorder
OSF: Open Science Framework
PANAS-X: Positive and Negative Affect Schedule – Expanded
PD: Panic Disorder
PTSD: Post Traumatic Stress Disorder
PS: Person-Stable (i.e., time invariant features)
RF: Random Forests
RMSE: Root Mean Squared Error
RNN: Recurrent Neural Network
SAD: Social Anxiety Disorder
SD: Standard Deviation
SP: Specific Phobia
