# Supplementary material for: Comparing personalized and population-based models for predicting momentary negative affect in internalizing disorders: A digital phenotyping study
Source: Neurosci Appl. 2026 Apr 28;5:107006. doi: 10.1016/j.nsa.2026.107006 (PMC13145394; doi:10.1016/j.nsa.2026.107006)
Supplement: Multimedia component 1 [file mmc1.docx]

**Supplementary Material**

**Supplementary Table 1. Overview of models and hyperparameters**

| **Pipeline** | **Base Model / Approach** | **Tested Hyperparameters** |
| --- | --- | --- |
| **Global_Intercept** | Population based intercept benchmark |  |
| **PerUser_Intercept** | Per Person based intercept benchmark |  |
| **LR** | Linear Regression | fit_intercept: [True, False] |
| **LR_with_PS** | Linear Regression | fit_intercept: [True, False] |
| **RF** | Random Forest Regressor | n_estimators: [50,100]; max_depth: [4,5] |
| **RF_with_PS** | Random Forest Regressor with person-stable features | n_estimators: [50,100]; max_depth: [4,5] |
| **FFNN** | Feed Forward Neural Network | hidden_units: [(64,32),(128,64),(128,64,32)];  batch_size: [32,64];  learning_rate: [1e-3,1e-4]; dropout_rate: [0.25,0.5] |
| **FFNN_with_PS** | Feed Forward Neural Network with person-stable features | hidden_units: [(64,32),(128,64),(128,64,32)]; batch_size: [32,64];  learning_rate: [1e-3,1e-4]; dropout_rate: [0.25,0.5] |
| **MERF** | Mixed Effect Random Forest | max_iterations: [10,15]; n_estimators: [50,100] |
| **MERF_with_PS** | Mixed Effect Random Forest with person-stable features | max_iterations: [10,15]; n_estimators: [50,100] |
| **FFNN_with_**  **Embeddings** | Feed Forward Neural Network with person-specific embedding layer | embedding_dim: [2,4,6]; hidden_units: [(64,32),(128,64),(128,64,32)]; batch_size: [32,64] |
